# Impact of the COVID-19 pandemic on acute mental health admissions in Croatia

**DOI:** 10.3389/fpubh.2023.1231796

**Published:** 2023-11-13

**Authors:** Karolina Kalanj, Marko Ćurković, Mirta Peček, Stjepan Orešković, Ante Orbanić, Rick Marshall

**Affiliations:** ^1^Department of Medical Oncology, Clinic of Oncology, Clinical Hospital Center, Zagreb, Croatia; ^2^Andrija Štampar School of Public Health, University of Zagreb School of Medicine, Zagreb, Croatia; ^3^University Psychiatric Hospital Vrapče, Zagreb, Croatia; ^4^School of Medicine, University of Zagreb, Zagreb, Croatia; ^5^Independent Consultant, Zagreb, Croatia; ^6^Epidemiologist and Independent Consultant in Health System Funding Models, Eaglehawk Neck, TAS, Australia

**Keywords:** COVID-19, data transparency, health system response, inpatient mental care, pandemic, patient classification system, psychiatry, diagnosis related group

## Abstract

**Background:**

The pandemic of COVID-19 had a profound impact on our community and healthcare system. This study aims to assess the impact of COVID-19 on psychiatric care in Croatia by comparing the number of acute psychiatric cases before coronavirus disease (2017–2019) and during the pandemic (2020–2022).

**Materials and methods:**

The paper is a retrospective, comparative analyzes of the hospital admission rate in Diagnosis Related Group (DRG) classes related to mental diseases, and organic mental disorders caused by alcohol and drug use. This study used DRG data from all acute hospitals in Croatia accredited to provide mental health care services and relevant publicly available data from the Croatian Institute of Public Health (CIPH) and the Croatian Health Insurance Fund (CHIF). All hospital admissions for acute psychiatric patients in Croatia were tracked during both periods under study.

**Results:**

During the pandemic, the average number of all such cases decreased by 28% in secondary and tertiary hospitals, and by 11% in specialist psychiatric hospitals. It was also found that during COVID-19, there was a decrease in case numbers in DRG classes related to major affective disorders and anxiety, alcohol, and drug intoxication (31, 48, 34 and 45%, respectively). However, the same period saw an increase in hospital activity for eating disorders and for involuntary admissions related to schizophrenia and paranoia (30, 34 and 39% respectively). There were no changes in the admission rate for cases related to opioid use.

**Conclusion:**

The COVID-19 pandemic resulted in both a steep decrease in the overall number of psychiatric cases inpatient treatment at mental health facilities and their DRG casemix. Increasing our understanding of how pandemics and isolation affect demand for psychiatric care will help us better plan for future crises and provide more targeted care to this vulnerable group.

## Introduction

COVID-19 was initially detected in Wuhan, Hubei Province, China, around the end of 2019. The World Health Organization declared COVID-19 a pandemic after it spread over continental boundaries and began to affect almost every nation by March 11, 2020 (1).

The initial wave of infection’s duration and severity was unknown, and there was genuine concern that hospitals would be overrun by COVID-19 patients who were extremely ill and in need of a specific kind of (critical) care and, therefore, would run out of space, resources, and personnel (2).

As a result of an increase in COVID-19 cases and mortality, European nations have implemented steps to control the pandemic and protect their healthcare systems. Depending on the country, these widespread public health interventions have included social distancing, border closures, school closings, steps to isolate symptomatic individuals and their contacts, and population lockdowns save for essential internal travel (3). On the one hand, this approach could be successful in achieving its primary objectives. Still, on the other hand, it might lead to feelings of isolation and solitude while also having a disruptive influence on formal and informal support and help networks. In addition, such exceptional and uncertain circumstances would tend to increase levels of anxiety, stress, or depressive symptoms in the community (4, 5).

Croatia’s approach to COVID-19 broadly mirrored other European countries’ strategies, while, according to the Government Stringency Index (GSI) its mitigation measures were initially rather restrictive (with GSI close to 100) while from late November 2020 might be considered as relatively mild (with GSI around 50 and later on 30).

In response, with the need to prioritize pandemic threats, health care facilities changed their infrastructure, processes, and personnel. In this vein, psychiatric facilities, services as well as personel were often repurposed, an occurence most notably expresed in significant downsizing or were complete reorganization of psychiatric inpatient units. Some of the units were changed from psychiatric to medical capacity, while others were retained vacant in preparation for expected future increases in patient demand for acute medical care or used for auxiliary functions (supply, operation centers, staff respite, etc.) (6). Such, trend was also witnessed in Croatia, where many psychiatric units within larger clinical hospitals were either drastically downsized or even completely repurposed.

Like other countries, even when psychiatric facilities remained fully operational, analyzes revealed a significant decrease in utilization, within first pandemic year, in both hospitalizations and outpatient visits.

For example, analysis of the impact of COVID-19 pandemic on activity of University Psychiatric Hospital Vrapče in Zagreb, the largest tertiary psychiatric institution in Croatia, revealed that inpatient care decline was most significant within the group of affective disorders (7) (interestingly enough, these patterns were observed during Spanish flu in 1918 as well and during the Homeland war in 1990’s) (8), but also significant in other clinical categories. More recently, it was reported that, at the same institution, simultaneously with general decrease of utilization of inpatient and regular outpatient psychiatric services in pandemic 2021 compared to prepandemic 2019, an increase in the utilization of emergency psychiatric services was observed (9).

To avoid exposure to pandemic threat, many people reduced or stopped doing their regular activities, including visiting hospitals or outpatient units. Such avoidance was most likely the source of the drop in the utilization of outpatient services. Major contributing causes to the lack of attendance at scheduled visits at outpatient units during the pandemic included older age, higher anxiety, lower confidence in coping with COVID-19 followed by greater commitment to preventive health behaviors, as avoiding crowded areas, wearing protective masks, and more often washing hands (10). Mental health services in Croatia are still mostly provided within institutions (with several notable exceptions, e.g., services for substance abuse disorders), although there is a stated, general tendency toward more community oriented services. It should be noted, that there is also a widespread lack of adequate child and adolescent mental health services.

Within this setting, COVID-19 pandemic introduced some major changes, or probably more accurately–accelerated already initiated ones, most pronounced in a shift toward greater share of outpatient services while especially those provided by the means of information and communication technologies (ICT). In Croatia also, similar to other settings, emergence of COVID-19 pandemic was accompanied by a rather early and widespread policy deregulation of “telehealth” services. By the end of the first pandemic year, first and central telepsychiatric institution in Croatia (at the University Hospital Vrapče) was fully established and recognized by all the relevant authorities.

Also, some more specific mental health projects took place (e.g., prevention of burnout in health care workers, public campaigns raising awareness of depression, development of “stress and antistress” (low threshold, stepped) outpatient programs, outreach to citizens impacted by earthquake effects.).

The aim of this study is to evaluate the direct impact of COVID-19 on mental health care admissions in all acute and special psychiatric hospitals accredited to provide inpatient services in Croatia, in the period before (2017–2019) and during the pandemic (2020–2022). The main objective of the research is to provide data for policy development to guide the formulation of effective measures to protect access to needed mental health care services at times when the health care systems come under stress. Importantly, the study demonstrates that routinely collected DRG data can be used to analyze the impact of COVID-19 on the provision of hospital mental health care at the population level. It presents findings that would otherwise require access to specific primary mental care admission data from all hospitals that provide such services.

## Materials and methods

### Study design and data sources

Data was gathered from databases maintained by the Croatian Institute of Public Health (CIPH) and the Croatian Health Insurance Fund (CHIF), both of which are accessible to the general public (11). The Croatian DRG system is based on the Australian Refined DRGs (AR-DRG system), utilizing a combination of the International Classification of Diseases and Related Health Problems, Tenth Revision Australian Modification (ICD-10 AM) and ICD-10 classifications for the coding diagnosis and the Australian Classification of Health Interventions (ACHI) for the coding procedures. The Croatian DRG grouping approach is based on the AR-DRG version 5.2 and acute cases may be classified into 671 DRG classes (12). The 23 Major Diagnostic Categories (MDC) essentially reflect the particular medical specialty based primarily on the main diagnose, which represents the main reason for patient being admitted in hospital. Each MDC corresponds to a certain body system or etiology, and the system is in line with the ICD10 classification. In this study, MDC19 and MDC20 groups were examined since they represent mental, behavioral, and neurodevelopmental disorders, alcohol, drug use, and organic mental disorders induced by their consumption (13). All hospital admissions for psychiatric patients in Croatia’s acute care hospital facilities and specialist psychiatric hospitals were tracked from the year 2017 to the year 2022. Overall, 22 secondary-level hospitals, 9 tertiary-level hospitals, and 8 special psychiatric hospitals were observed.

Based on the reason for the patient’s admission, each episode of care was classified by CHIF into its appropriate DRG group related to mental health, and as a result, changes in acute patient admission before and during the COVID-19 pandemic were observed. Our study did not need informed permission or ethical approval because the data used were completely anonymized and made accessible as public information from CHIF and the CIPH, which were subject to Croatian patient data protection rules.

### Data and statistical analysis

Three years (2017–2019) before the pandemic and 3 years (2020–2022) during the pandemic were used to compute the average number of inpatient cases for DRGs related to mental health. The incidence admission rate (IR) for each DRG group (not a single mental disorder) was then calculated by dividing the average number of cases throughout the particular period by the average total population based on the Croatian Bureau of Statistics population estimates for 2017–2019 and 2020–2022. The incidence admission rate difference for each DRG group between each of the two periods was then divided by the incidence admission rate in the preceding period to arrive at the % change in incidence admission rate. The incidence admission rate between the two periods was compared using the 2-by-2 Chi-square test. As a ratio of two rates (2017–2019 and 2020–2022), the incidence admission rate ratio (IRR) for each analyzed DRGs was calculated. Based on an analysis of whether the IRR was equal to one, the Wald technique was used to compute the 95% confidence intervals. Microsoft Excel was used to calculate average values and rate change, while R (R Core Team, Austria) was used to run every statistical analysis (14). As data were compared by calendar year, a constant variance was assumed, and therefore no adjustments for seasonal effects and autocorellation were not needed. Statistical significance was defined as a value of p of 0.05 or less.

## Results

The results from this retrospective data analyzes were presented in three subsections below: (a) the admission rate change for all inpatient psychiatric cases before and during pandemic and per hospital type (tertiary, secondary and special psychiatric hospitals); (b) the admission rate change for episodes of care grouped in V–DRG group Alcohol, Drug Use and Alcohol/Drug Induced Organic Mental Disorder; (c) the admission rate change for episodes of care grouped in U–DRG group (conditions related to Mental, Behavioral and Neurodevelopmental Disorders).

The average number of acute psychiatric patients in all hospitals during the pandemic (2020–2022) was 18,716, of which 5,285 (28,24%) were treated at the tertiary health care level, 5,960 (31,84%) at the secondary health care level, and 7,471 (39,92%) were treated at special psychiatric hospitals. In comparison to pre-pandemic period (2017–2019), the average number of acute psychiatric patients in all hospitals was 24,005 of which 7,382 (30,76%) were treated at the tertiary health care level, 8,224 (34,26%) at the secondary health care level, and 8,399 (34,99%) were treated at psychiatric hospitals. The overall rate change for the observed hospital network is −22% (*p* < 0.0001), the decline for tertiary and secondary hospitals is −28% (*p* < 0.0001) while for special psychiatric hospitals is −11% (*p* < 0.0001; Table 1; Supplementary Table S1).

**Table 1:** Comparison of total hospital admissions during pre-pandemic (2017–2019) and pandemic (2020–2022) period.

	Average number of admissions (2017–2019)	Average number of admissions (2020–2022)	Admission rate change	Value of *p*
Tertiary hospitals	7,382	5,285	−28%	<0.0001
Secondary hospitals	8,224	5,960	−28%	<0.0001
Special psyhiatric hospitals	8,399	7,471	−11%	<0.0001
Total	24,005	18,716	−22%	<0.0001

Source: CHIF and authors calculation.

During the pandemic, there were 4,481 patients admitted because of conditions related to the V-code DRG group, that is, conditions related to Alcohol, Drug Use and Alcohol/Drug Induced Organic Mental Disorder.^1^ Compared to the pre-pandemic period, there is an average drop of 22% (*p* < 0.0001), when the total number of patients was 5,733. During the pandemic, 1,127 (25,15%) patients were treated at the tertiary health care level, 1,317 (29,39%) at the secondary, and 2,038 (45,48%) at special psychiatric hospitals. The number of patients dropped significantly (*p* < 0.0001), −20% at the tertiary, −29% at the secondary health care level, and − 17% at the special psychiatric hospitals.

In order to calculate the difference between number of admissions during prepandemic and pandemic period, the average number of admissions was calculated for each V DRG codes, and decrease was observed for the following DRG groups: V60A (−30%; *p* = 0,1,191), V60B (−34%; *p* < 0.0001), V61Z (−45%; p < 0.0001), V62A (−23%; *p* < 0.0001) and V62B (−37%; *p* = 0,0817). A decrease less than calculated average of 22% for all V DRG codes admissions was observed in V63B (−6%; *p* = 0,7,569), and in V64Z (−13%; *p* = 0,0747). However, the difference in number of admissions for V60A, V62B was higher than average of 22% for all admissions but not statistically significant and the reason may be a small number of admissions in those groups in both observed period. An increase of 68% in V60A and 224% in V63A was observed at the tertiary health care level, but when data for the same DRG groups were analyzed for all hospitals no statistically significant difference was found (p 0,1191 and p 0,9850 respectively; Table 2).

**Table 2:** Comparison of total hospital admissions during pre-pandemic (2017–2019) and pandemic (2020–2022) period for V-Code DRG group.

	2017–2019			2020–2022			2017–2019	2020–2022	Prepandemic-pandemic comparison	Prepandemic-pandemic comparison	Prepandemic-pandemic comparison	Prepandemic-pandemic comparison	*p*-value
Codes	T	S	PS	T	S	PS	Average all hospitals	Average all hospitals	% Admission Rate change (T)	% Admission Rate change (S)	% Admission Rate change (PS)	% Admission Rate change (All)	
V60A	6	15	26	11	14	9	47	33	68%	−11%	−65%	−30%	0.1191
V60B	247	389	853	244	235	505	1,489	984	−1%	−39%	−41%	−34%	<0.0001
V61Z	205	56	78	67	49	71	339	187	−67%	−13%	−9%	−45%	<0.0001
V62A	801	1,220	1,226	579	889	1,031	3,247	2,500	−28%	−27%	−16%	−23%	<0.0001
V62B	11	22	3	9	8	5	36	23	−21%	−62%	78%	−37%	0.0817
V63A	29	38	91	93	21	43	157	157	224%	−44%	−53%	0%	0.9850
V63B	25	20	15	25	19	12	60	56	1%	−7%	−16%	−6%	0.7569
V64Z	89	92	175	97	80	133	356	310	8%	−13%	−24%	−13%	0.0747

T, Tertiary hospitals; S, Secondary hospitals; PS, Psychiatric hospitals; Source: CHIF and authors calculation.

Table 2 compares the average number of total admissions during the pre-pandemic (2017–2019) and pandemic years (2020–2022) for conditions related to Alcohol, Drug Use and Alcohol/Drug Induced Organic Mental Disorder (V-Code DRG group) in all three groups of hospitals.

**Figure 1:**
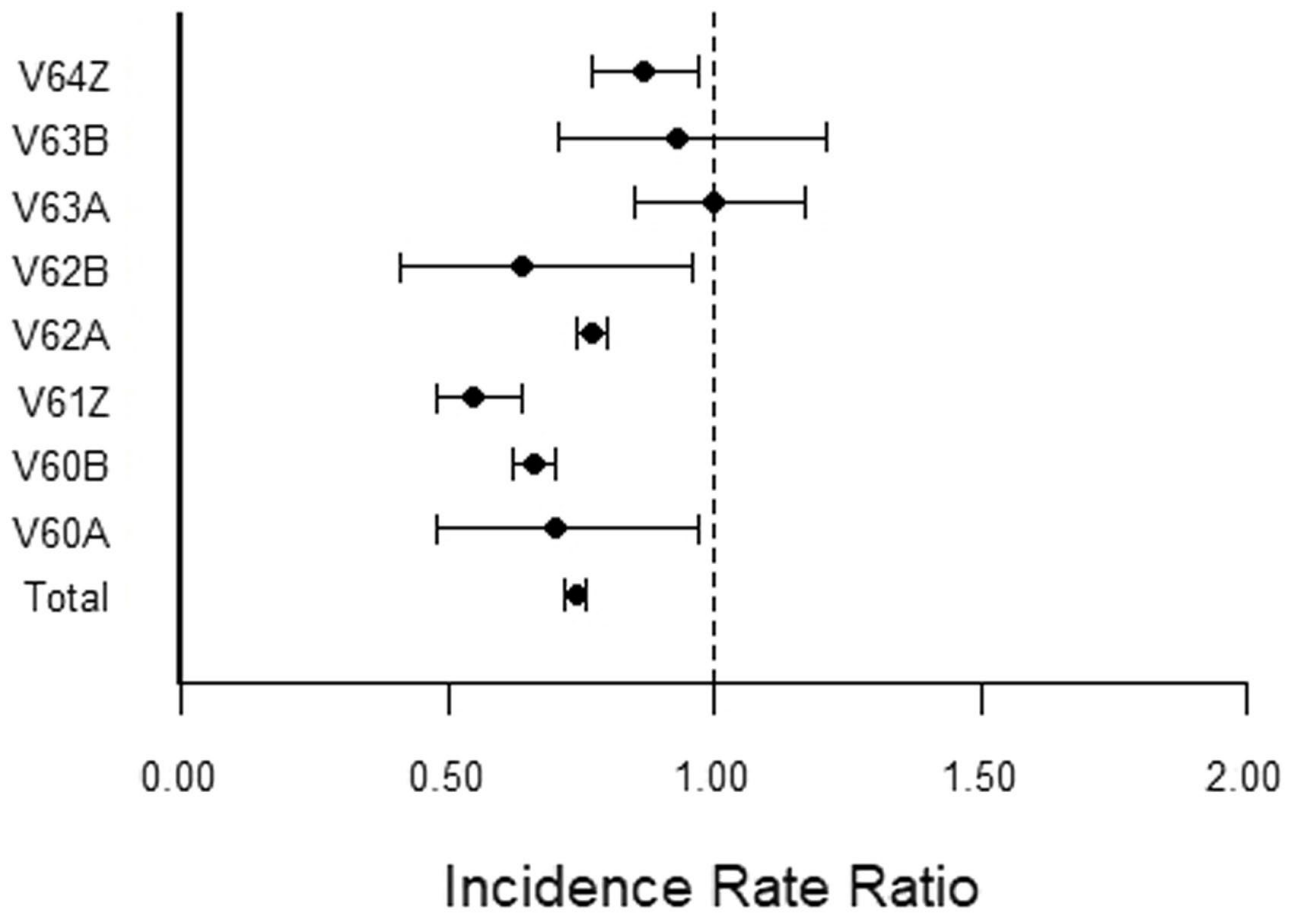
Incidence rate ratio (IRR) for conditions related to Alcohol, Drug Use and Alcohol/Drug Induced Organic Mental Disorder (V-Code DRG group) during the pandemic (2020–2022) compared to pre-pandemic (2017–2019).

Figure 1 shows the corresponding IRRs calculated for conditions related to Alcohol, Drug Use and Alcohol/Drug Induced Organic Mental Disorder (V-Code DRG group).

During the pandemic, there were 14,235 patients treated because of conditions related to the U-code DRG group, that is, conditions related to Mental, Behavioral and Neurodevelopmental Disorders.^2^ Compared to the pre-pandemic period, there is an average drop of 22% (*p* < 0.0001), when the total number of patients was 18,272. During the pandemic, 4,158 (29,21%) patients were treated at the tertiary health care level, 4,643 (32,62%) at the secondary, and 5,434 (38,17%) at special psychiatric hospitals. The number of patients dropped significantly (*p* < 0.0001), −30% at the tertiary, −27% at the secondary health care level, and − 8% at the special psychiatric hospitals.

The decrease in the number of admissions during pandemic period greater than average number of admissions during prepandemic period was observed in U61B (− 23%; *p*  < 0.0001), U63A (−27%; *p* < 0.0001), U63B (−31%, *p* < 0.0001), U65Z (−48%; *p* < 0.0001), and U67Z (−26%; *p* < 0.0001). A decrease less than average was observed in U60Z by 10% (*p* = 0,5,318), U62B by 13% (*p* < 0.0001), and U64Z by 18% (*p* = 0,0012). An increase was observed in U61A by 34% (*p* = 0,0668), U62A by 39% (*p* = 0,0454), U667 by 30% (*p* = 0,0140), and U68Z by 17% (*p* = 0,0017; Table 3).

**Table 3:** Comparison of total hospital admissions during pre-pandemic (2017–2019) and pandemic (2020–2022) for U-Code group in all three groups of hospitals.

	2017–2019			2020–2022			2017–2019	2020–2022	Prepandemic-pandemic comparison	Prepandemic-pandemic comparison	Prepandemic-pandemic comparison	Prepandemic-pandemic comparison	*P*-value
Codes	T	S	PS	T	S	PS	Average all hospitals	Average all hospitals	% Admission Rate change (T)	% Admission Rate change (S)	% Admission Rate change (PS)	% Admission Rate change (All)	
U60Z	30	28	21	18	27	26	79	71	−39%	−6%	27%	−10%	0.5318
U61A	27	19	24	26	21	45	69	93	−2%	13%	92%	34%	0.0668
U61B	1,505	1800	2,139	1,069	1,350	1792	5,444	4,211	−29%	−25%	−16%	−23%	<0.0001
U62A	24	21	19	31	23	35	64	88	31%	10%	79%	39%	0.0454
U62B	692	926	670	592	731	669	2,288	1993	−14%	−21%	0%	−13%	<0.0001
U63A	149	205	75	100	145	68	429	312	−33%	−29%	−10%	−27%	<0.0001
U63B	1,456	1,557	1,016	856	1,097	846	4,029	2,799	−41%	−30%	−17%	−31%	<0.0001
U64Z	174	247	144	101	189	172	565	461	−42%	−24%	19%	−18%	0.0012
U65Z	129	144	258	97	93	87	530	276	−25%	−35%	−66%	−48%	<0.0001
U66Z	108	17	27	135	19	44	152	198	25%	14%	62%	30%	0.0140
U67Z	1,265	1,389	1,255	872	912	1,094	3,908	2,878	−31%	−34%	−13%	−26%	<0.0001
U68Z	407	15	285	257	18	555	707	830	−37%	15%	95%	17%	0.0017

T, Tertiary hospitals; S, Secondary hospitals; PS, Psychiatric hospitals; Source: CHIF and authors calculation.

An increase of 79% acute admissions in DRG groups U62A and 92% in U61A was observed during the pandemic at special psychiatric hospitals.

Table 3 compares the average number of total admissions during the pre-pandemic (2017–2019) and pandemic years (2020–2022) for conditions related to Mental, Behavioral and Neurodevelopmental Disorders (U-Code DRG group).

**Figure 2:**
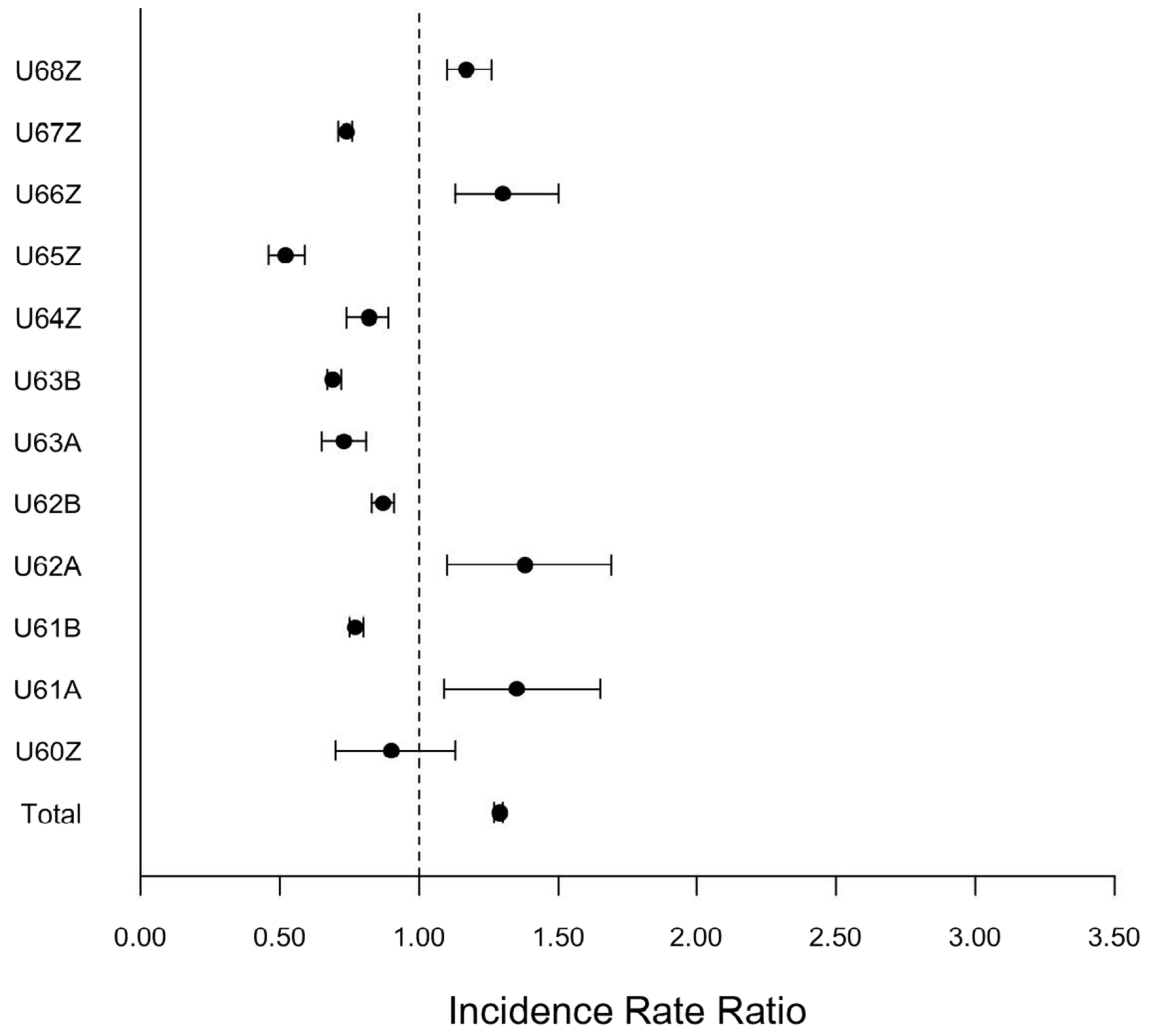
Incidence rate ratio (IRR) for corresponding IRRs calculated for conditions related to Mental, Behavioral and Neurodevelopmental Disorders (U-Code DRG group) during the pandemic (2020–2022) compared to pre-pandemic (2017–2019).

Figure 2 shows the corresponding IRRs calculated for conditions related to Mental, Behavioral and Neurodevelopmental Disorders (U-Code DRG group).

## Discussion

It has been reported that due to many interlinked reasons mental health burden of disease has been severely impacted since the outbreak of the pandemic. The severe reduction in human interaction, forced solitude, the deconstruction of the everyday activities, and the loss of active and recognized social roles have all led to an increase in subjective psychological fragility, making it impossible to restart a fulfilling existence (15, 16).

The utilization pattern of hospitals in Croatia during the COVID crisis period showed trends that are consistent with both: a change in patient needs for inpatient care settings (for a few DRG classes admission rates increased) and a shift in hospital intake protocols in response to the pandemic demand management priorities and emergency restrictions.

During the pandemic, there was a significant average decrease in the total number of admissions and in the number of hospitalized acute psychiatric patients at both secondary (−28%) and tertiary (−28%) hospital levels, as well as at special psychiatric hospitals (−11%). We determined the same average drop of 22% for both observed U-code and V-Code DRG groups.

The initial COVID-19 patient was confirmed in Croatia on February 25th, 2020. Three weeks later, in response to an increased COVID-19 patient load and an increased risk of contagion, hospital care delivery was changed to accommodate the pandemic’s assessed needs. Three hospitals in Zagreb, the capital city of Croatia, were recognized as COVID-19 centers, and patients with COVID-19-related diseases and complications needing inpatient care were admitted to those institutions. Most hospitals created COVID-19 isolation wards, and four similar facilities were created across the country (17).

In addition to the reorganization of the healthcare system, in 2020 stronger social restrictions (lockdown) were also witnessed. Elective procedures being given lower priority by hospitals, a decline in the non-emergency admission referral rate due to fewer outpatient hours, and a scarcity of hospital staff are contributing factors. Fear of obtaining COVID-19 infection in a healthcare setting reported by patients is another (18).

Furthermore, in March 2020, Zagreb faced another disaster - a devastating earthquake that severely affected ability of some hospitals to provide a full range of health care services and had a great impact on mental health (19, 20).

The initial limited studies published in the literature appear to imply an unexpected drop in absolute terms of Emergency Department (ED) admissions for an acute psychiatric conditions in both the US and Europe in the first 6 months of COVID-19 pandemic (21, 22). Some of the causes for this drop might include home isolation with increasing difficulties in contacting health services, fear of infection, and fear of discriminating behaviors, which rose during the epidemic (23). However, the drop in ED visits does not appear to be solely due to psychiatric conditions, since the general decline in ED visits reported at the pandemic’s early commencement appears to be between 40 and 70% (24, 25).

A comprehensive cross-sectional research in the United States that took into consideration period till October 2020 found that with the start of the pandemic, visits for psychiatric conditions increased in proportion to the fall in overall ED visits. The rates of ED visits for a mental disorders, suicide attempts, and drug or opioid overdoses were above the 2019 average rates and remained higher throughout the research period, mainly due to a large spike in March 2020 (26). However, considering only the first few months of the pandemic may not be enough to provide definitive portrayal about the impact of SARS-CoV-2 on acute psychiatric pathology, as it is well known that crises and disasters can cause an increase in mental health issues not only in the short term but especially in the long term (27, 28).

Di Lorenzo et al. reported that between March 1, 2020, and August 31, 2020, the number of requests for an urgent psychiatric visit in the ED reduced from 602 to 476, in comparison with the same period in 2019. Nevertheless, in 2020, a substantial rise in patients referred to the ED from psychiatric inpatient facilities was observed, owing to a rise in serious conditions that were unmanageable, such as suicidal conduct or maladaptive states with anxiety and violence (29).

According to Capuzzi et al., 225 emergency psychiatric consultations were done in the Lombardy region during the first wave of the COVID-19 pandemic, slightly more than half (58%) of the similar time in 2019 (388 emergency department visits). Residence permit in a mental hospital, cannabis dependency, and obsessive-compulsive disorder (OCD) diagnosis were all statistically significant predictors of emergency psychiatric consultation during lockdown measures (30).

Similar findings were observed at a metropolitan hospital in Portugal, with a quick drop in psychiatric ED visits occurring within 2 weeks of the emergency state pandemic period (31) and within a month at a big tertiary hospital in Connecticut (32). However, Goncalves-Pinho and colleagues also observed that ED visits increased consistently after the first 2 weeks (31). Yet, there were variances in the relative decreases for each category, with schizophrenia and other psychotic illnesses experiencing the smallest decline (9,8% compared to the 2019 era) and mood disorders experiencing the greatest relative decrease (68,3%) compared to the 2019 period (31).

In this study, we found that schizophrenia disorders without need for involuntary admission experienced an average decline of 23%, and schizophrenia disorders with involuntary admission experienced an average increase of 34%. This is consistent with results published by Fasshauer et al. using a large inpatient sample from 13 HELIOS hospitals in Germany (total n = 64,502) that the proportion of urgent and involuntary admissions for all psychiatric diagnoses significantly increased in 2020 as compared to 2018 (*p* < 0.001) and 2019 (*p* < 0.0001) (33).

Persons with schizophrenia and other psychotic diseases are, in ordinary circumstances, a considerable part among group of patients hospitalized for psychiatric conditions. Also, such patients can, by the nature of disorder and its wanning and waxing course, require urgent psychiatric care, as for example, in the cases of intensification of hallucinations, thought or behavioral disorganization, or delusions, which all can also lead to manifestion of various forms of aggression (34). Furthermore, such patients might also lack awarenes of their disorder (and thus might be more relactant to seek help), while involvement of family members or even application of certain involuntary measures that direct them to necessary services might be required.

Nonetheless, observed average increase of more complex (acute) schizophrenia cases during pandemic period might be driven by different factors. On the one hand, such findings might signal that more complex and more severe patients with schizophrenia and other psychotic disorders might have greater difficulties in adapting to novel, exceptional, uncertain circumstances, such as COVID-19 pandemic. On the other hand, in such circumstances, usual, formal and informal support structures and networks, both health and non-health related ones, have been seriously impacted (while, assumably, more complex and more severely impaired patients might be even more vulnerable to its adverse effects). Both of those factors might contribute to a greater probability of mental decompensation to such an extent that urgent (or even a prolonged one) inpatient care is needed. One additional set of factor was previously discussed in literature could be of importance here, a more direct effects of SARS-CoV-2 infection, as it may also increase symptoms in persons with schizophrenia, as coronaviruses have been linked to psychotic symptoms via an immune-related mechanism (35).

It is important to note that this observed increase is mostly driven by the increase of cases within special psychiatric hospitals (where also decrease among less complex cases was present). This might be reflective of the fact that those services were less affected by COVID-19 related health care system reorganizations (that were more prominent in psychiatric facilities within other health care institutions/hospitals).

Devoe et al. discovered a strong influence on the rise in the number of hospitalizations due to eating disorders (EDs) following the commencement of the pandemic in their systematic review of the effects of the pandemic on patients with EDs. The pooled average of 11 studies found a 48% increase in hospital admissions during the pandemic compared to the same period the previous year, with an 83 and 16% rise in pediatric and adult admissions, respectively (36). We found a 30% increase in group related to EDs and obsessive compulsive disorders (OCD), also being most notable in special psychiatric hospitals, a somewhat more unusual occurrence than within that observed in schizophrenia group as, in principle, patients with EDs require a more comprehensive and holistic consideration of both physical and mental health, especially in urgent situations. Nontheless, this is probably also reflective of pandemic induced changes within health care system.

The overall COVID-19 pandemic setting was a particularly fertile ground for the development and perpetuation of various paranoid ideas, beliefs, and even delusions, as, by default of “new normal,” literally everyone was perceived as threat while cues and following attribution processes were markedly hindered (f.e. by masking or lack of social exposure). Evenmore, an upsurge of paranoia is generally associated with actual, real uncertainties. Some previous studies suggest that such rise of paranoia was less significant in states that imposed a more aggressive lockdown and more pronounced upon reopening in states that required mask-wearing. The pandemic’s early phase in 2020 heightened people’s paranoid ideas and beliefs and made their updates more irregular. This was especially noticeable in states where mask-wearing restrictions were not strictly enforced, but where rule compliance was more widespread. Unsurprisingly, sources and related contents of paranoid thoughts were increasingly more COVID-19 specific, while more paranoid ones were more prone to believe in mask-wearing conspiracies and possible immunizations (37). In our analyzes, increase and decrease of paranoia and acute psychotic disorders cluster mirrored those of schizophrenia with an average rise of 39% in more complex group related to involuntary admission U62A (again driven by the contribution of special psychiatric hospitals), and notable decrease within less complex group U62B Group (within psychiatric units by secondary and tertiary level hospitals).

If taken together, findings concerning all psychotic disorders (schizophrenia, paranoia, and acute psychotic disorders), it seems to be more accurate to interpret those findings in terms of significant overall decrease, in the utilization of the acute inpatient psychiatric services, in pandemic when compared to pre-pandemic period.

Considering the impact of the COVID-19 pandemic on mental health disorders in children, the review by Bai et al. reported higher levels of anxiety, depression, post-traumatic stress disorder, and sleep disorders in children compared to the pre-pandemic period (38). We also observed a rise of 17% in childhood mental disorders group.

As seen in the aftermath of economic crises (39), terrorist attacks (40), and natural catastrophes (41), major negative events and crises impacting society can generate changes in alcohol usage at the population level (42). Similarly, the spread of and reactions to SARS-CoV-2 may have led to changes in alcohol usage, with concerns raised regarding prospective increases in drinking levels in particular (43). Alcohol is a key contributor to the global illness burden (44), and despite recent decreases, Europe has the greatest *per capita* consumption in the world, with three out of every five people using alcohol (45).

Two primary explanations have been proposed to explain variations in alcohol consumption during the pandemic. The first mechanism relates to higher levels as a result of both prevention strategies and the danger of personal exposure to COVID-19 or the sickness of a loved one (42, 43). Psychological stress and anxiety are established risk factors for excessive alcohol use and are thus likely to boost consumption during the COVID-19 pandemic (46). The second mechanism, on the other hand, predicts a decrease in alcohol usage as a result of reduced availability and cost of alcoholic drinks during this period, as well as a decrease in possible drinking opportunities as a result of actions aimed at reducing social gatherings (39). Alcohol policy research provides evidence for this mechanism, highlighting lower availability and cost of alcoholic drinks as effective policy measures to reduce alcohol consumption at the population level (47). Both of these processes might have had a significant effect in promoting variations in alcohol consumption during the pandemic (48). We observed that average number of admissions during pandemic time was lower when compared to the average number of admissions during prepandemic period in all DRG groups related to alcohol consumption: V60A (*p* = 0.1191), V60B (*p* < 0.0001), V62A (*p* < 0.0001), V62B (*p* = 0,0817).

Interestingly enough, the reduction in number of admissions during pandemic was also observed within group of disorders that are more oftenly and clearly associated with unfavorable external conditions and circumstances, that is, major affective disorders (DRG groups U63A and U63B), anxiety disorders (DRG group U65Z) and personality disorders and acute reactions (DRG group U67Z). Finally, one could expect significant increase exactly in those mental health maladies and conditions. However, it seems that a rather consistent (meta)findings from many studies aimed at exploring COVID-19 influences on mental health–despite known, expected and often observed clearly negative effects of overall pandemic setting on mental health, such negative effects failed to materialize in various “hard” outcomes, as for example, suicides (49, 50) or as reported here, in the utilization of acute inpatient psychiatric services. The reasons underlying such occurences are still not well understood.

More optimistic interpretations highlight timely and robust emphasis that has been placed on mental health from the early pandemic and expansion of quantity, reach, and kinds of outpatient services, especially those mediated by information and communication technologies. Also, a specific psychological mechanisms have been proposed as playing an important role, such as proximity of death experiences, collective nature of the threat, *et cetera*. In addition, it might be that we just consistently underestimate strenghts and, more generally, resilience of our societies as well as its agencies involved in the provision of health care services.

More pessimistic outlooks propose materialization of a more serious and devastating mental health consequences in a longer terms (that is, are yet to come), with the decisive additional effects of the ongoing “livelihood crisis.”

Some views even highlight the possibility that psychiatric institutions were likely (unnecesarily) overutilized in prepandemic times, due to various, often non-psychiatric in a strict sense, reasons. Following these lines of thought, one could argue that all the pandemic pressures may have moved psychiatric services toward a more realistic and sustainable balance. However, this might be somewhat at odds with general and global pleas toward a more subtle, finely graded, comprehensive, continous, inclusive, accountable and person-centered mental health services.

Finally, findings presented here, taken together with many others we rely upon in discussion, quite certainly re–emphasize - the complexity of mental health disorders. Not only the complexity of its causes, but also representations, and corresponding patterns of help seeking and providing care. Here again, it seems that public (as opposed to private), shared (as opposed to individual) and structurly organized (as opposed to incidental) factors, quite certainly, have a more decisive roles and influences.

### Strengths and limitations

Our study is the first national complete study using all inpatient psychiatric cases in Croatia in the pre-pandemic and pandemic periods. As the Croatian health care system is based on universal health insurance coverage and medical services related to mental health care are exempted from copayment, the study findings are not biased due to differential access to providers within the observed periods.

The limitation of the study are two–fold. The first is an aggregate nature of DRG data used and analyzed, that might obscure some rather specific intra-group/cluster dynamics, as we did not analyze admissions per specific diagnoses. Also, DRG data in Croatia are used for payment of hospital services, there is a risk of the main diagnose being miscoded, but without access to patient level data it is impossible to exclude or reassign those cases in appropriate DRG groups. The second concerns our inability to investigate impact of COVID-19 on outpatient mental health care provided by the observed hospital network.

## Conclusion

To our knowledge, this is the first study that shows the impact of COVID-19 pandemic on inpatient care for patients with psychiatric conditions related to acute mental health care in Croatia. We observed a significant average decrease in the total number of admissions and in the number of hospitalized acute psychiatric patients at both secondary and tertiary hospital levels, as well as at special psychiatric hospitals. Our data imply that the COVID-19 pandemic resulted in a quick drop in the emergency department and inpatient treatments at mental health facilities. This might be due to a complicated interaction between patients (e.g., a greater threshold for coming to the ED and seeking admission) and clinicians (e.g., a higher threshold to admit). Many of the changes in pandemic-related patient and provider behavior might be understood as logical responses to the rebalancing of risk–benefit assessments for seeking or delivering mental care during a pandemic. Recent research suggests that this decline in mental treatment usage may have long-term repercussions. Improving our understanding of how COVID 19 pandemic influenced mental care usage may help us to prepare for future crises and offer better, more integrated care for this vulnerable group.

## Data availability statement

Publicly available datasets were analyzed in this study. This data can be found at: www.cezih.hr.

## Author contributions

KK, MĆ, and MP led design of the study. KK, AO, and SO organized the data collection and analysis of the first draft. RM and AO performed the statistical analysis of the data collected and wrote the findings section. All authors contributed to the development of the research question, study design in relation to the Croatian DRG patient classification system, interpretation of the results and critical revision of the manuscript for the important intellectual content. All authors contributed to the article and approved the submitted version.

## Footnotes

^1^Vcodes: V60A-Alcohol Intoxication and Withdrawal W CC; V60B-Alcohol Intoxication and Withdrawal W/O CC; V61Z-Drug Intoxication and Withdrawal; V62A-Alcohol Use Disorder and Dependence; V62B-Alcohol Use Disorder and Dependence, Sameday; V63A-Opioid Use Disorder and Dependence; V63B-Opioid Use Disorder and Dependence, Left Against Medical Advice; V64Z-Other Drug Use Disorder and Dependence.

^2^U codes: U40Z-Mental Health Treatment, Sameday, W ECT; U60Z-Mental Health Treatment, Sameday, W/O ECT; U61A-Schizophrenia Disorders W Mental Health Legal Status; U61B-Schizophrenia Disorders W/O Mental Health Legal Status; U62A-Paranoia & Acute Psych Disorder W Cat/Sev CC or W Mental Health Legal Status; U62B-Paranoia & Acute Psych Disorder W/O Cat/Sev CC W/O Mental Health Legal Status; U63A-Major Affective Disorders Age > 69 or W (Catastrophic or Severe CC); U63B -Major Affective Disorders Age < 70 W/O Catastrophic or Severe CC; U64Z-Other Affective and Somatoform Disorders; U65Z-Anxiety Disorders; U66Z-Eating and Obsessive-Compulsive Disorders; U67Z-Personality Disorders and Acute Reactions; U68Z-Childhood Mental Disorders.
